# Comparative Evaluation of Compressive and Diametral Tensile Strength in Die Stone Reinforced With Different Types of Nanoparticles: An In Vitro Study

**DOI:** 10.7759/cureus.41131

**Published:** 2023-06-29

**Authors:** Aman Ali, Abhishek Gaur, Kaushik Kumar Pandey, Shaily Tyagi, Fauzia Tarannum, Mohd Azeem, Mohd Umar, Neha G Khatuja, Shyamolima Hazarika, Manjari Shrivastava

**Affiliations:** 1 Prosthodontics, Career Post Graduate Institute of Dental Sciences and Hospital, Lucknow, IND; 2 Prosthodontics, Saraswati Dental College, Lucknow, IND; 3 Prosthodontics, Santosh Dental College, Ghaziabad, IND

**Keywords:** die stone, universal test machine, type iv dental stone, diametral tensile strength, compressive strength, nanoparticles

## Abstract

Aim: To investigate the effect of different types of nanoparticles on the compressive strength (CS) and diametral tensile strength (DTS) of type IV dental stones.

Materials and methods: A total of 100 specimens were made from the mould for all five groups. Four commercially available nanoparticles (aluminium oxide (Al_2_O_3_), silicon dioxide (SiO_2_), zinc oxide (ZnO), and zirconium oxide (ZrO_2_)) were used in this study in a concentration of 10%. CS and DTS tests were performed in a universal test machine. The data were statistically analysed using ANOVA and Student's t-test.

Results: The interaction between nanoparticles and the type of dental stone was found to be statistically significant (p < 0.05). CS and DTS values decreased by adding all four nanoparticles. The lowest CS and DTS were observed in 10% ZnO nanoparticles when added to type IV dental stone.

Conclusion: It was concluded that the addition of nanoparticles (Al_2_O_3_, SiO_2_, ZnO, and ZrO_2_) to die stone significantly decreased the CS and DTS for all groups. Among all groups, the incorporation of 10% ZrO_2 _nanoparticles (group E) to die stone showed significantly less decrease in CS and DTS compared to Al_2_O_3_, SiO_2_, and ZnO. Incorporation of ZnO nanoparticles, on the other hand, showed a significantly more amount of decrease in the CS and DTS compared to Al_2_O_3_, SiO_2_, and ZrO_2_.

## Introduction

Gypsum products are one of the most broadly utilized dental materials for the fabrication of dental casts and die, which are then used for further construction of indirect dental restorations. Gypsum products are obtained from natural gypsum minerals. The American Dental Association (ADA) classified gypsum products into five different types according to their properties and uses, which are impression plaster (type I), dental plaster (type II), dental stone (type III), dental stone high strength (type IV), and dental stone, high strength/high expansion (type V) [1].

Owing to their premium mechanical properties, such as great resistance to abrasion and high compressive strength (CS), type IV improved stone is commonly used in the fabrication of dental casts. Also, it exhibits high accuracy when compared with other gypsum product types due to its little setting expansion [2].

A strong cast with smooth and hard surface characteristics is necessary for the ease of wax sculpting, particularly at the cervical margins without any cast abrasion. Since the cavity preparation is filled with wax that is carved by flushing with the margins of the die, it is mandatory for the die stone to have a hard surface. The selection of stone depends on mechanical properties, such as surface roughness, diametral tensile strength (DTS), CS, wear resistance, surface hardness, and ability to reproduce the detail. The CS and DTS have been the most prevalent laboratory testing procedures to distinguish between the mechanical and physical properties of dental stones [2].

The strength of dental materials has now been improved with the application of inorganic filler particles. Quartz, colloidal silica, and silica glass containing barium, strontium, and zirconia are among the various types of inorganic filler particles that are available these days. With the incorporation of filler particles, the properties of the materials can be affected owing to the shapes and sizes of filler particles [3]. To improve the properties of flexibility, strength, plasticity, mechanical compatibility, and biocompatibility, nanomaterials are used with ceramic, metal, resin, and composite materials. This further helps in the reduction of porosity in the materials and makes their modulus of elasticity similar to the form of natural bone. There have been recent studies on the effect of adding different types of functionalized nanoparticles to polymethyl methacrylate (PMMA) and evaluating the effect of these particles on PMMA properties after adding different shapes, sizes, and, ratios of them [4].

However, the effect of the incorporation of nanoparticles on the mechanical properties of type IV gypsum products has not been established yet. Hence, this study was planned to evaluate and compare the mechanical properties (CS and DTS) of type IV gypsum products after the addition of different types of nanoparticles.

## Materials and methods

In the present study, type IV improved dental stone (Ultrarock, Kalabhai Karson Pvt. Ltd., Mumbai, India) was used as a gypsum material. Aluminium oxide (Al_2_O_3_), silicon dioxide (SiO_2_), zinc oxide (ZnO), and zirconium oxide (ZrO_2_) nanoparticles (99.9% pure, particle size 30-50 nm, Adnano Technologies, Majjigenahalli, India) were used as reinforcing materials. The sample size for the study was determined after discussing with the statistician before carrying out the research. A total of 100 specimens were fabricated. The samples were equally divided into five groups (groups A, B, C, D, and E) and each group had 20 samples. Ten samples were used for measuring CS and 10 for measuring DTS (Table 1).

**Table 1:** Sample distribution

Group	Specimen group	No. of specimens for compressive strength	No. of specimens for diametral tensile strength	Total specimens
A	Without nanoparticles (Control group)	10	10	20
B	Containing 10% wt. Al_2_O_3 _nanoparticle	10	10	20
C	Containing 10% wt. SiO_2 _nanoparticle	10	10	20
D	Containing 10% wt. ZnO nanoparticle	10	10	20
E	Containing 10% wt. ZrO_2_ nanoparticle	10	10	20
	Total specimens	50	50	100

**Figure 1:**
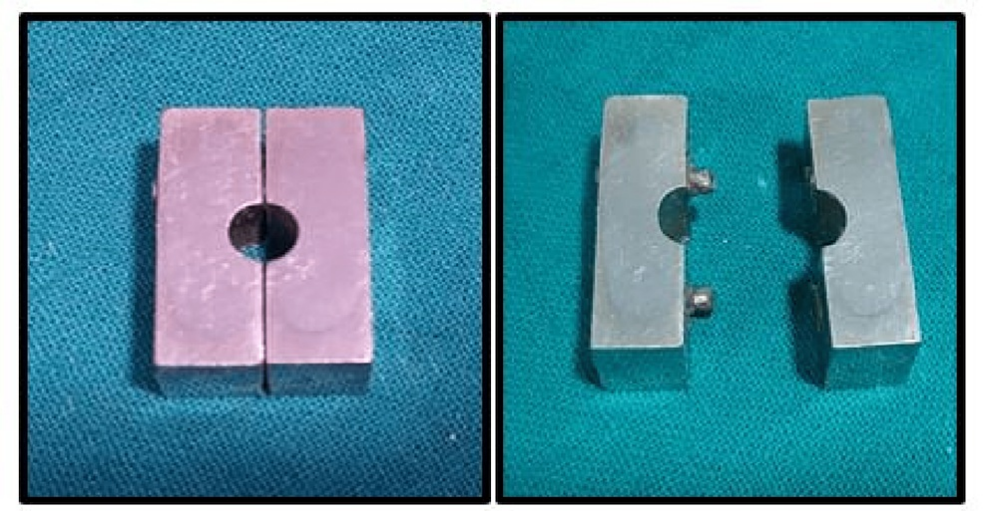
Customized mould of dimension 7 x 14 mm for compressive strength

**Figure 2:**
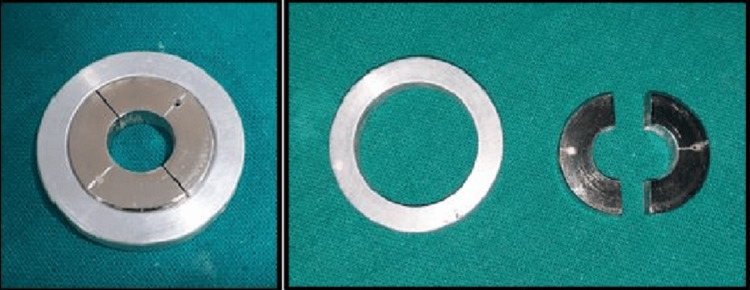
Customized mould of dimension 14 x 7 mm for diametral tensile strength

The customized mould (stainless steel) was fabricated of a specific dimension with a diameter of 7 x 14 mm in the ratio of 1:2 (width:height) of the mould space for CS (mould A) and for DTS, the diameter of 14 x 7 mm in the ratio of 2:1 (width:height of the mould space B), according to ISO 6873 (Figures 1, 2).

**Figure 3:**
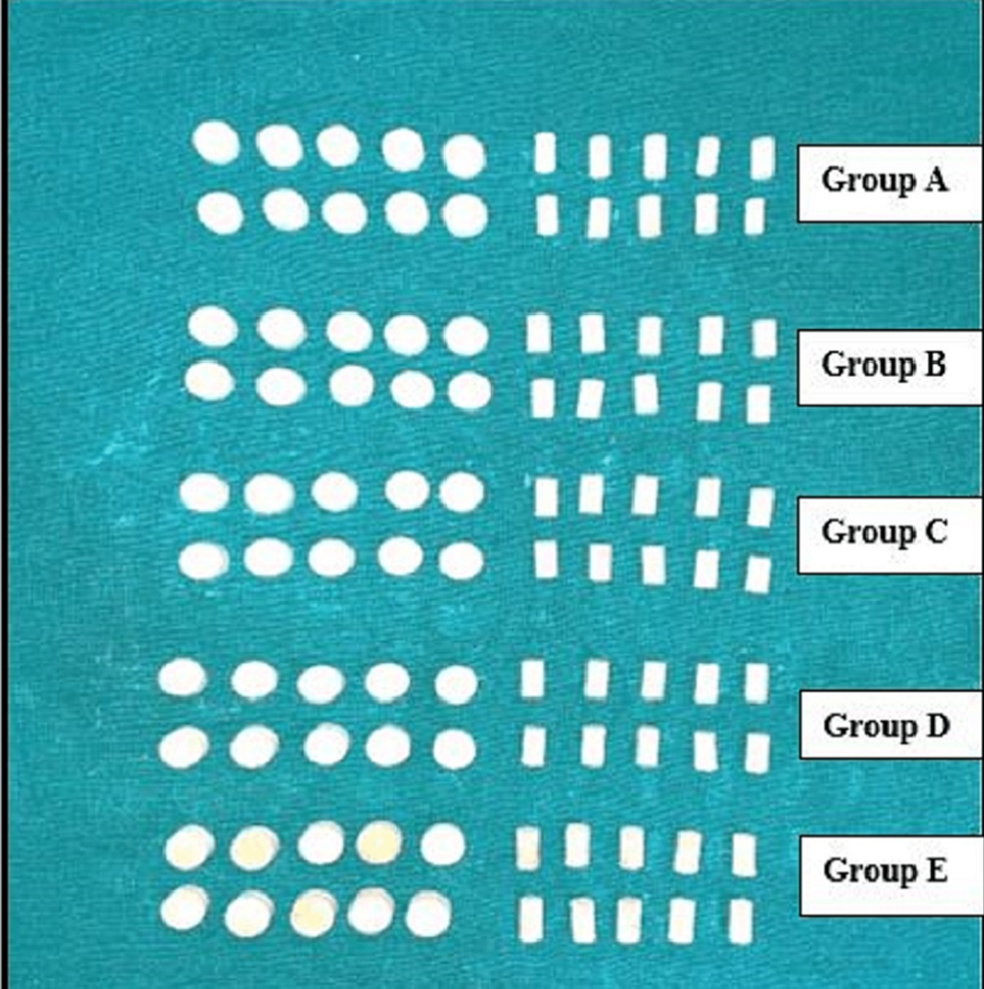
Specimen samples of all groups

A total of 100 specimens were made from the mould for all the five groups. Fifty were made from mould A to test for CS and 50 were made from mould B to test for DTS (Figure 3). For the CS test, the force is applied along the long axis of the specimen whereas, and in DTS, the specimen is diametrically compressed introducing tensile stress in the plane of the force of action of the specimen. Hence, two different moulds were fabricated. During the fabrication of each specimen, both the moulds were coated with petroleum jelly (Vaseline, Hindustan Unilever Ltd., Haridwar, India), which helped in the easy removal of the specimen from the mould.

The die stone powder and nanoparticle powder were weighed using a digital scale (Anamed, MX Series, Ahmedabad, India) and distilled water was measured using a 10 ml glass pipette (BFC, Glasscolabs, Ambala, India), as recommended by the manufacturer and according to the test group for the nanoparticles. Nanoparticles were mixed with die stone manually. The die stone was mechanically spatulated under a vacuum mixer (Elite Mix, Confident Dental Equipments Ltd., New Delhi, India) following the time recommended by the manufacturer and poured into the mould under vibration. The specimens were allowed to be set for one hour before separating the moulds. Glass plate was placed on the bottom and top of the mould to obtain specimens with flat surfaces.

To standardize the procedure, the manufacturer's instructions were followed. All standard equipment and instruments were used. The specimens were carefully retrieved from the mould, air-dried for one hour, and stored in an airtight container. Mechanical tests were then performed in a digital universal testing machine (Central Institute of Plastic Engineering and Technology, Lucknow, India).

**Figure 4:**
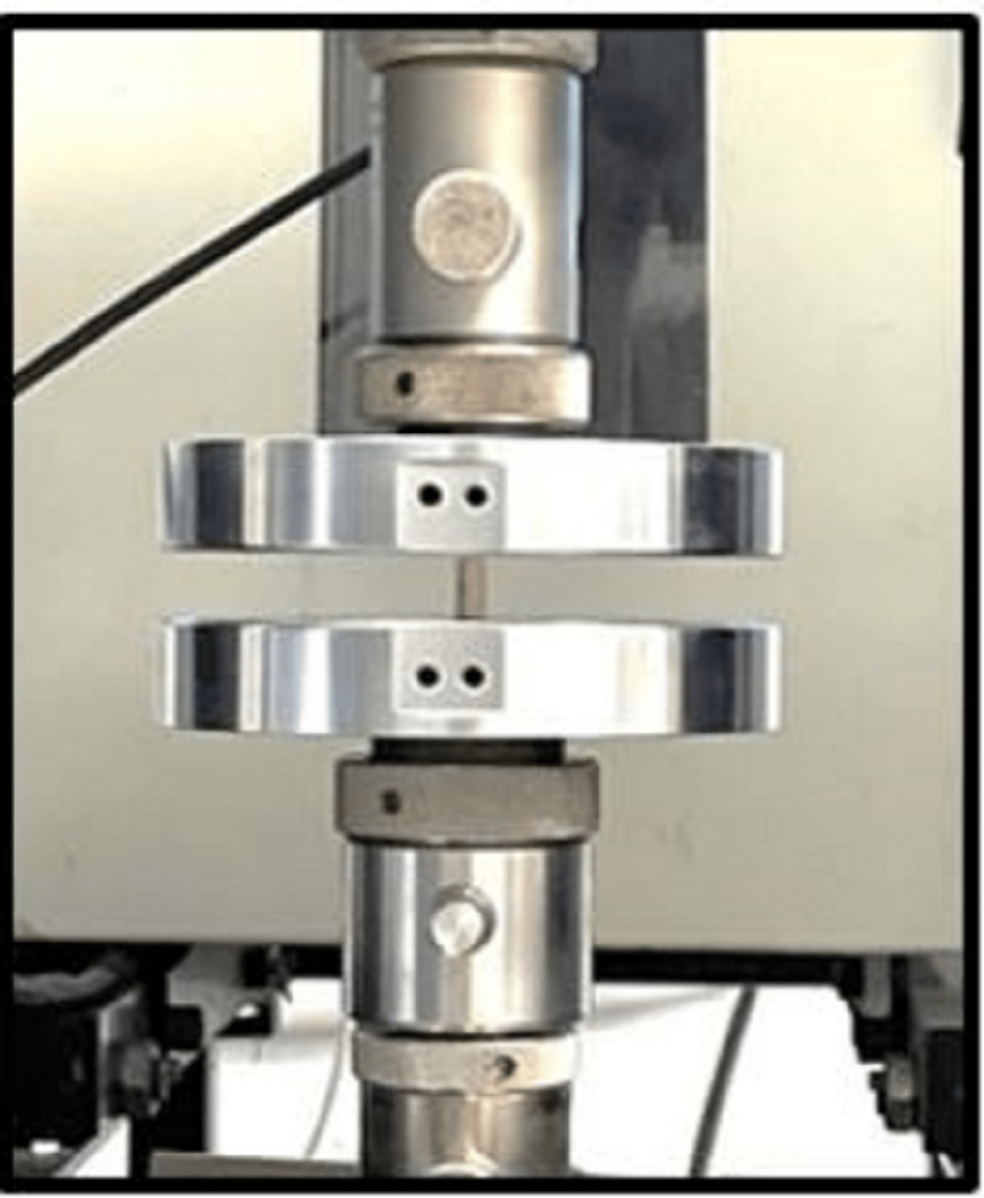
Specimens tested for compressive strength in a digital universal testing machine

The selected specimens were stored for 24 hours under room temperature conditions for mechanical tests to be performed. The test procedure was employed using standard instruments on the prepared specimen. The test specimens were tested on digital universal testing machine at a cross-head speed of 1 mm/minute [2]. The specimens were placed with flat ends between the plates of the apparatus, so that load will be applied at the long axis of the specimen. Compressive tests were performed in a universal test machine with 1 mm/minute cross-head speed. Compressive loading was applied until the specimen was broken and compressive load values were recorded (Figure 4). CS values were calculated by the following formula [5]: CS = load (N)/area (cm2). Surface area = area of the circle × 3.14 cm.

**Figure 5:**
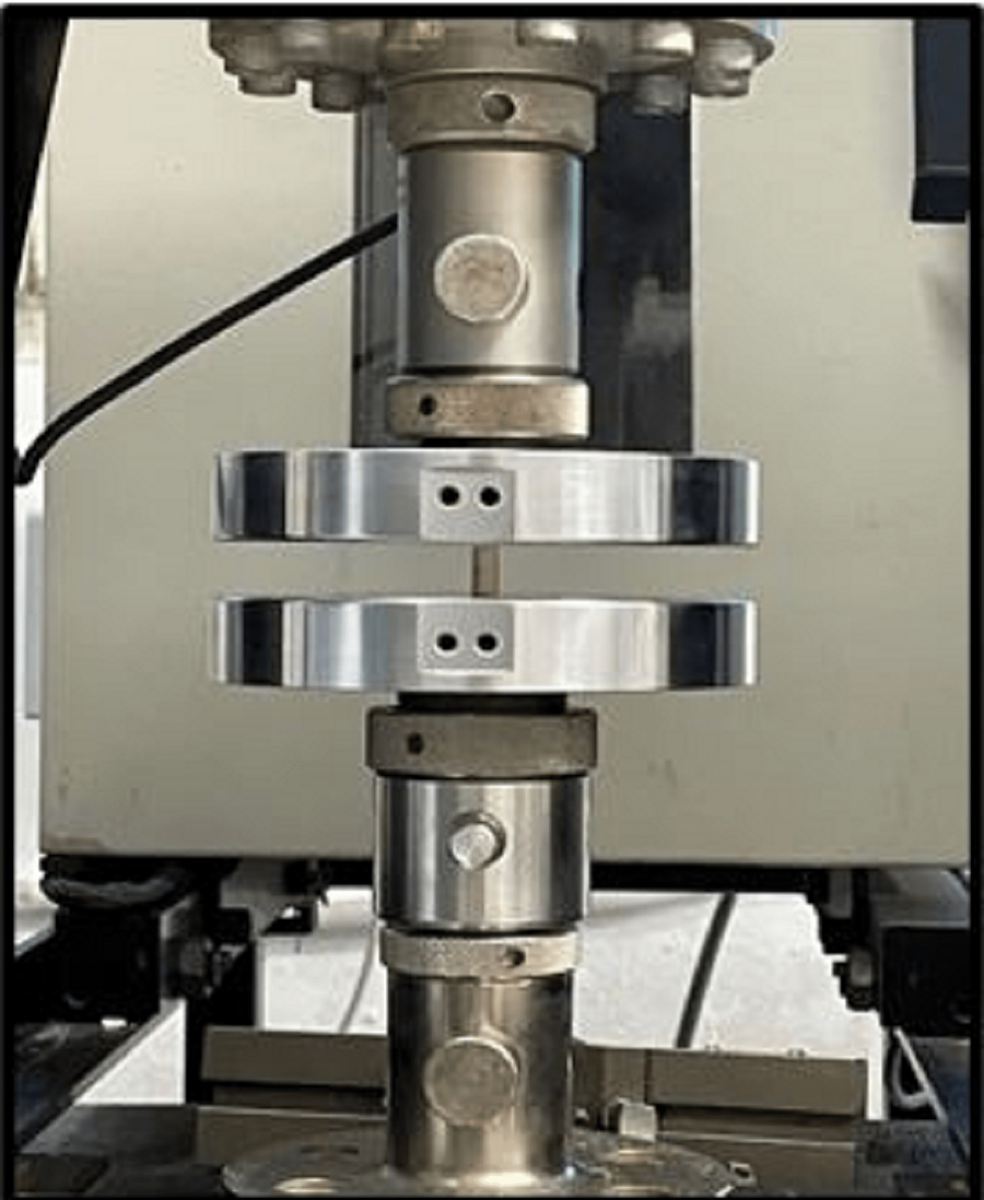
Specimens tested for diametral tensile strength in a digital universal testing machine

Diametral tensile tests were performed in a universal test machine with a 1 mm/minute cross-head speed (Figure 5). DTS was calculated by the following formula [5]: DTS = 2P/π × D × T, where P is the load, D is the specimen diameter, and T is the specimen thickness.

## Results

The entire study data were evaluated using an independent t-test and ANOVA test with the help of statistical software (SPSS version 13.0 for Windows, SPSS Inc., Chicago, IL) [2].

On the evaluation of mean CS, it was found that mean CS was maximum in group A (21.5740 ± 0.46085), group B (5.9540 ± 0.38067), group C (2.1110 ± 0.29471), group D (1.2310 ± 0.34491), and group E (6.8730 ± 0.52135) in MPa units. On intergroup comparison, with the help of an independent t-statistical analysis test, highly statistically significant results were found (P < 0.001), which revealed that group A (control group) had significantly higher mean value as compared to all other groups (B, C, D, and E). A maximum mean difference was observed between group A (control group) and group D (ZnO nanoparticle), and a minimum group difference was observed between group A and group E (ZrO2). On the basis of the above observations, the order of CS in different groups was as follows: group A > group E > group B > group C > group D (Table 2).

**Table 2:** Intergroup comparison of compressive strength *** Very highly statistically significant (P < 0.001), ** highly significant (P < 0.01), significant (P ≤ 0.05), and insignificant (P > 0.05). The mean value is expressed in MPa. The statistical test used for analysis was the independent t-test.

Strength		Mean (MPa)	Std. deviation	Std. error mean	P-value
Compressive strength (MPa)	Group A	21.5740	0.46085	0.14573	<0.001**
	Group B	5.9540	0.38067	0.12038	
	Group C	2.1110	0.29471	0.09320	
	Group D	1.2310	0.34491	0.10907	
	Group E	6.8730	0.52135	0.16486	

On the evaluation of mean DTS, it was found that mean DTS was maximum in group A (24.0520 ± 0.25403), group B (6.329 ± 0.11881), group C, (2.4430 ± 0.70413), group D (2.1460 ± 0.35839), and group E (15.1190 ± 0.53288) in MPa units. On intergroup comparison of DTS with the help of an independent t statistical analysis test, highly statistically significant results were found (P < 0.001), which revealed that group A (control group) had significantly higher mean value as compared to all other groups (B, C, D, and E). A maximum mean difference was observed between group A (control group) and group D (ZnO nanoparticle), and a minimum group difference was observed between group A and group E (ZrO2). On the basis of the above observations, the order of DTS in different groups was as follows: group A > group E > group B > group C > group D (Table 3).

**Table 3:** Intergroup comparison of diametral tensile strength *** Very highly statistically significant (P < 0.001), ** highly significant (P < 0.01), significant (P ≤ 0.05), and insignificant (P > 0.05). The mean value is expressed in MPa. The statistical test used for analysis was the independent t-test.

Strength		Mean (MPa)	Std. deviation	Std. error mean	P-value
Diametral tensile strength (MPa)	Group A	24.0520	0.25403	0.08033	<0.001**
	Group B	6.3290	0.70413	0.22267	
	Group C	2.4430	0.35839	0.11333	
	Group D	2.1460	0.11881	0.03757	
	Group E	15.1190	0.53288	0.16851	

On comparison of CS and DTS of type IV stone (within group and between group), highly significant (P < 0.001) results were found. ANOVA test was used for this statistical analysis (Table 4).

**Table 4:** Compressive strength and diametral tensile strength between and with all groups *** Very highly statistically significant (P < 0.001), ** highly significant (P < 0.01), significant (P ≤ 0.05), and insignificant (p > 0.05). The unit used is MPa. The statistical test used is the ANOVA test.

Strength	Groups	Sum of square (MPa)	df	Mean square	F	P-value
Compressive strength	Between groups	2683.713	4	670.928	4017.946	<0.001
	Within groups	7.514	45	0.167		
Diametral tensile strength	Between groups	3588.102	4	897.025	4544.847	<0.001
	Within groups	8.882	45	0.197		

## Discussion

Die materials play a crucial role during the fabrication of indirect dental restorations and prostheses. Die materials with the highest quality in terms of accuracy and strength are highly recommended for indirect methods of fabrication of inlays, crowns, and bridges [6].

Non-gypsum die materials such as acrylic resin and polyester have been available for some time. These materials are limited in their compatibility with the impression materials and, because of a high curing contraction, the accuracy of the die is affected. Epoxy die materials appear to be reliable with respect to dimensional changes in polymerization. Although when these materials are used, it may be necessary to adjust the investing and casting procedures [7].

During the process of hardening, a model and die material should have minimal expansion and excellent strength. Due to its superior mechanical properties such as CS, hardness, and low expansion properties when compared to other gypsum products, type IV die stone is thus widely used for the fabrication of dies and master casts for fixed and removable partial prostheses [5,8].

The criteria used for the selection of stone include its mechanical properties such as surface roughness, DTS, CS, wear resistance, surface hardness, and ability to reproduce the detail [6]. For the differentiation of mechanical and physical properties of dental stone, CS and DTS have been the most common laboratory testing modalities [9].

The implementation of nanotechnologies has rapidly developed in all areas of healthcare science, including odontological science [10]. Dental materials make use of different types of inorganic fillers, including particles such as quartz, colloidal silica, and silica glass containing barium, strontium, and zirconia. These filler particles having different shapes and sizes are used in commercial products and have an effect on the properties of the materials [11]. An important and recent change in inorganic fillers has been the application of nanotechnology to the development of dental products, with the main goal of improving their mechanical properties [12].

Nanoparticles have been rendered to be an effective medium for various dental applications due to their unique properties, which include their surface-to-volume ratio, antibacterial action, physical, mechanical, and biological characteristics, and unique particle size [13]. Though a wide variety of nanoparticles are available in the literature, nanoparticles such as aluminium oxide (Al_2_O_3_), zirconium oxide (ZrO_2_), titanium dioxide (TiO_2_), zinc oxide (ZnO), silicon dioxide (SiO_2_), and silver (Ag) have often been used nowadays [14].

In the present study, 10% of Al_2_O_3_, SiO_2_, ZnO, and ZrO_2_ nanoparticles were added separately in die stone to evaluate and compare the CS and DTS. The results revealed a significant decrease in CS and DTS. This was in accordance with the study conducted by De Cesero et al. [15]. The reason for the reduction in CS and DTS may be attributed to the decrease in inter-crystallization cohesion between the gypsum crystals, which may lead to an increase in the concentration of additives in stone materials. Another factor could be a slight increase in the water-powder ratio during mixing thereby creating pores inside the material that weaken it due to the presence of fewer crystals by volume [15].

A similar study done by Akkus et al. [2], where Al_2_O_3_ and SiO_2_ were used as reinforcing nanoparticles, also resulted in a decrease in CS and DTS of type IV stone, which was in accordance with our study. These findings could be attributed to the use of nanoparticles and also to the changes in the water/powder ratio recommended by the manufacturer. Another study conducted by Salah et al. [16] to evaluate the CS using ZnO nanoparticles as a reinforcing material had similar results as observed in our study. It was found that as the concentration of ZnO nanoparticles was increased, the CS value decreased. A similar study done by Salah et al. [17] using Ag nanoparticles as a reinforcing material had findings that were in similarity to our study.

Taqa et al. [18] in their study evaluated the CS and surface hardness of dental stone (type III) by the addition of 0.5%, 1%, 1.5%, and 2% concentrations of rosin, Nigella sativa oil, and sodium lauryl sulphate (different chemical materials) in contrast to the addition of nanoparticles used in our study. The results found in their study showed an increase in the CS and surface hardness of dental stones. A study conducted by Khalaf et al. [19] was also comparable to the findings observed in our study where they made use of 1% silver nitrate powder for treating type IV dental stone specimens with disinfecting powders. The decrease in strength could be attributed to some of the hemihydrates crystals that did not hydrate to form the dihydrate crystals because of an increase in the rate of reaction. On the other hand, the results found in our study were in contrast to a study conducted by Kati et al. [20]. In their study, instead of nanoparticles, certain additives like cured resin, pulverized plaster, and glass fibres and drying methods (air and microwave) were used to investigate the CS of dental plaster and stone. It was found that the CS was higher after the incorporation of these additives.

In the present study, it is evident from our findings that CS and DTS of type IV dental stones were decreased by the addition of Al_2_O_3_, SiO_2_, ZnO, and ZrO_2 _nanoparticles in a 10% ratio. Thus, the addition of these nanoparticles in type IV stone would not be recommended in this particular ratio since it has led to a decrease in both the CS and DTS. Thus, further research needs to be done either by changing the percentage of nanoparticles, types of nanoparticles, techniques of mixing of nanoparticles, or incorporation of nanoparticles after performing thermal and mechanical ageing process so that favourable results could be obtained.

Limitations of the study

In this study, hand mixing was used for the incorporation of nanoparticles with the die stone, and wt.% of nanoparticles was added more than in previous research studies, so this could be one of the reasons for the decrease in CS and DTS when compared with that of the control group A (without nanoparticle). The lack of standardization of DTS methodology in the literature made it difficult to compare the results. Since thermal and mechanical ageing processes were not performed, it is not known whether these differences in the values would be observed in the laboratory use of type IV dental stones. The lack of these factors may be a limitation in our study and thus needs further research.

## Conclusions

From the present study, it was concluded that with the addition of 10% nanoparticles (Al_2_O_3_, SiO_2_, ZnO, and ZrO_2_) to die stones, the CS and DTS significantly decreased for all groups. In comparison with group A (control group), a highly significant decrease was found in the CS and DTS in all other groups (B, C, D, and E). Among all the groups, the incorporation of 10% ZrO_2_ nanoparticles (group E) to die stone showed significantly less decrease in CS and DTS compared to Al_2_O_3_, SiO_2_, and ZnO. The incorporation of ZnO nanoparticles, on the other hand, showed a significantly more amount of decrease in the CS and DTS compared to Al_2_O_3_, SiO_2_, and ZrO_2_.
